# Depression and quality of life in cancer survivors: is there a relationship with physical activity?

**DOI:** 10.1186/1479-5868-4-65

**Published:** 2007-12-17

**Authors:** Nancy Humpel, Donald C Iverson

**Affiliations:** 1 Eastern Australia Dementia Training Study Centre, University of Wollongong, NSW, Australia; 2 Faculty of Health & Behavioural Sciences, University of Wollongong, NSW, Australia

## Abstract

**Background:**

Evidence is growing on the benefit of physical activity to improve well-being following a cancer diagnosis. This study examined changes in physical activity from pre to post diagnosis and explored this relationship with quality of life and depression.

**Methods:**

Participants were recruited by posters and by letter of invitation. The questionnaire was completed by 59 prostate and 32 breast cancer survivors.

**Results:**

Physical activity decreased by 72 minutes per week from pre to post diagnosis, although 20.9% reported having increased activity post diagnosis. Over 30% were considered depressed. Breast cancer participants who increased physical activity post diagnosis reported higher scores for Physical Wellbeing subscale (26 versus 21; F[1,29] = 5.19, p < .03), Emotional Wellbeing subscale (22 versus 19; F[1,30] = 4.57, p < .04) and Functional Wellbeing subscale (26 versus 19; F[1,30] = 9.03, p < .001). A greater proportion of participants taking part in no physical activity were depressed (55.6%; χ^2 ^= 6.83, p < .04).

**Conclusion:**

Over 25% of participants identified with emotional and/or well being problems, and more than half reported insufficient physical activity to yield benefits. Future research needs to gain a better understanding of why cancer survivors decrease their physical activity following a cancer diagnosis and what is necessary for them in order to retain or increase their physical activity.

## Background

A diagnosis of cancer and its subsequent treatments bring in its wake the almost certain probability of experiencing side effects which, in turn, results in a reduction in quality of life (QOL). Depression, anxiety, fatigue and sleep disturbance are among the most commonly reported problems experienced by cancer survivors [1-4]. For example, a recent review reported prevalence rates up to 38% for major depression and 58% for depression spectrum syndromes [5]. As expected, the prevalence rates vary depending on the criteria used to define depression, the type of cancer and the stage at diagnosis [5,6]. There is an increasing body of evidence supporting the contribution of physical activity in the prevention and treatment of mental health problems that are common in cancer survivors, including depression and anxiety [7,8].

The presence of side effects has the potential to impact overall quality of life (QOL) both during and following treatment. Ganz and colleagues [9] reported that women with breast cancer had a positive QOL one year post-treatment, but experienced a deterioration in the following two years in areas such as body image and sexual interest and functioning. It is interesting to note that reduced QOL in women with breast cancer has been found more than five years after treatment [10]. Low to moderate QOL has also been reported by 40–45% of prostate cancer patients [11] and in men following a prostatectomy, depression was not found associated with QOL [12].

In the past decade, interest in using physical activity to help alleviate some of the side effects from cancer and its treatments has gained momentum. The link between physical activity and health has been well established in the general population with regular physical activity having been demonstrated to reduce the burden from coronary heart disease, hypertension, type 2 diabetes, osteoporosis, and some cancers [13]. As an adjuvant treatment following cancer, regular physical activity has been shown to positively impact on the side effects from the cancer and its treatments, and thereby improve quality of life both during and following the treatment phase [14]. A growing number of cross sectional and intervention studies have shown physical activity is associated with improved physical and functional capacity and reduced fatigue after treatment [15,16]. Reviews of physical activity intervention programs for cancer survivors have demonstrated a range of physical and psychological benefits among participants [17-19].

The ultimate benefit would be the demonstration of an increased survival rate with physical activity. Such a relationship was first reported by Holmes and colleagues [20] who found that increased physical activity post diagnosis of breast cancer resulted in improved survival. A survival benefit occurred among women with hormone-responsive tumors who engaged in 1–3 MET hours of physical activity per week with a large benefit (RR of 0.50) occurring among women who expended between 9–14.9 MET hours per week (equating to walking three to five hours per week at an average pace). Recently two studies by Meyerhardt and colleagues [21,22] involving women with stage I to III colorectal cancer [22] and men and women with stage III colon cancer [21] also indicated an association between physical activity and increased survival rates. A significant survival benefit occurred among women with stage I to III colorectal cancer who expended ≥18 MET hours of physical activity per week (adjusted hazard ratio of 0.39 for colorectal cancer-specific mortality), and among men and women with stage III colon cancer who engaged in 18–26.9 MET hours per week (adjusted hazard ratio of 0.51 for disease-free survival). These studies indicate that relative reductions in mortality of 50–60% can be achieved through regular high-dose physical activity.

While the benefits of physical activity for cancer survivors are becoming increasingly evident and even compelling, we know surprisingly little regarding how a cancer diagnosis impacts on the level of physical activity from pre diagnosis to post diagnosis to post treatment. Reductions in physical activity during cancer treatment followed by an increase in physical activity post treatment, but not back to pre diagnosis levels, have been reported by breast and colorectal survivors [23,24]. Survivors who engaged in physical activity during treatment and those who maintained physical activity following treatment completion, reported better QOL than those who ceased or reduced physical activity. Survivors who failed to reinitiate physical activity after completing their treatment reported the lowest QOL one to four years later [23,24].

Irwin and colleagues examined physical activity data from 812 breast cancer patients who participated in the HEAL (Health, Eating, Activity and Lifestyle) study [25]. Total physical activity (in hours per week) declined by 3.8% among participants with in situ breast cancer, by 13.8% among those with Stage I breast cancer and by 8.9% among those with Stage II and IIIa breast cancer. The greatest decreases involved moderate and vigorous-intensity physical activities, as well as sports and recreational physical activities. The greatest decreases in physical activity levels occurred among participants who had multiple treatment modalities and who had Body Mass Index (BMI) levels >29.9. Overall, approximately 55% of participants reported decreasing their physical activity levels in the pre-post diagnosis time period. In a follow-up study involving HEAL participants Irwin [26] reported that 68% gained body weight and 74% gained body fat over the two year period starting from about 6 months post diagnosis.

There is a need for further studies to examine the patterns of change in physical activity pre to post diagnosis and amongst other populations of cancer survivors. There is also a need to examine these changes in activity levels in relation to psychological outcomes. Thus, the two main aims of this study were: 1) to examine changes in the physical activity levels of breast and prostate cancer survivors from pre cancer diagnosis to post diagnosis, and whether the changes were related to overall QOL as well as depression; and 2) to explore whether current physical activity levels were associated with QOL and depression. Whilst not the main purpose of this study, we also examined the relationship of changes in physical activity levels with body mass index (BMI).

## Methods

### Participants and procedure

This was a cross-sectional study of breast and prostate cancer survivors from Illawarra area in NSW, Australia. Posters and flyers about the project were placed in waiting rooms at an oncology day centre, a regional cancer care centre and in urologists' rooms. In addition, urologists mailed an invitation letter with a study flyer to all newly diagnosed prostate cancer patients. The recruitment materials asked interested patients to contact the research team by telephone. This study was approved by the University Human Research Ethics Committee and written consent was obtained from all participants. Participants attended an interview where they completed the questionnaire or they could return the questionnaire by mail. Participants were not screened for level of physical activity prior to inclusion in the study as participants with a range of activity levels were desired for comparison on outcome variables.

### Measures

Physical activity (PA) was measured by the Godin Leisure-Time Exercise Questionnaire [27,28]. Three questions asked about the frequency of mild, moderate and strenuous physical activity performed each week. The scale was adapted such that participants were also asked about the average number of minutes for each physical activity session. This questionnaire has been reported to have acceptable levels of reliability and validity [29]. Participants were asked to report, retrospectively, on a usual week's physical activity in the 6 months pre diagnosis, and for the current previous week. This scale has been used previously in cancer populations [23,30,31].

Quality of life was evaluated using the Functional Assessment of Cancer Therapy system (FACT-G) which measures four aspects of quality of life: physical, functional, emotional and social well-being. Two additional aspects ask about concerns specific to breast and prostate cancer. The psychometric properties of the FACT scales are well documented [32,33] and have been found appropriate for use in cancer clinical trials. In this study the subscales had the following internal consistencies: physical (PWB; α = .82), social (SWB; α = .83), emotional (EWB; α = .64), functional wellbeing (FWB; α = .86), breast additional concerns (α = .76) and prostate additional concerns (α = .61); and for global FACT-G (α = .86).

Depression was measured by the Centre for Epidemiological Studies-Depression scale (CES-D) which is a validated and reliable 20-item self-report measure of depression that has been used in many populations, including cancer patients [34,35]. Participants are asked to indicate how they felt during the past week. Response options ranged from 'rarely/none of the time' to 'most of the time'. A cut-off score of 16 is used as an indication of depression. Body Mass Index (BMI) was calculated from participants' height and weight.

### Data analysis

Repeated measures t-tests were used to compare mean minutes of physical activity pre and post diagnosis. The minutes of strenuous, moderate and low physical activity were summed to give the total physical activity minutes. Scores on PA minutes were highly skewed with a large proportion of participants reporting no activity; as a result using PA minutes as a continuous variable was deemed unsuitable. Physical activity levels were further categorised into: those reporting no physical activity at all; those participating in some physical activity but not enough to meet the national physical activity guidelines; and those meeting the physical activity guidelines of greater than 150 minutes of physical activity each week [36,37]. To represent overall change in PA between the pre to post diagnosis periods, two categories were created: (1) participants whose weekly physical activity minutes either decreased or did not change; (2) participants whose weekly physical activity minutes increased. Analysis of variance was used to examine differences in quality of life and depression by demographics. Chi square analyses were used to examine relationships amongst categorical variables. Logistic regression was used to explore whether any of the variables under investigation predicted change in physical activity.

## Results

Table 1 includes participant demographic data by cancer type and percent of participants who had satisfied the guideline of engaging in >150 minutes of PA per week. The participants (n = 91) included 32 females who had a diagnosis of breast cancer and 59 males with a prostate cancer diagnosis. The mean age of all participants was 61 years (SD = 10.5), with the mean age for the breast and prostate cancer participants, respectively, being 53 (SD = 10) and 65 (SD = 7) years. Only 21% of prostate cancer participants had a tertiary education compared to 50% of the breast cancer participants. Most of the participants were married (79%) and were within one year of diagnosis (71%). Prostate cancer participants, breast cancer participants <51 years, prostate cancer participants ≥66 years, breast cancer participants with a tertiary education comprised the subgroups most likely to have achieved the recommended level of >150 minutes of PA per week with at least 50% of the participants in these groups having met this standard. However, there were no significant differences in the proportion of participants achieving the recommended standard of >150 minutes per week of PA by age, education level or cancer site.

**Table 1:** Demographics by cancer site and proportion reporting sufficient physical activity post diagnosis.

	Breast % (n)	Prostate % (n)	Sufficient PA % (n)
Men		64.8 (59)	39.0 (23)
Women	35.2 (32)		18.8 (6)
			
Age			
<50	50.0 (16)	5.2 (3) **	15.8 (3)
51–65	25.0 (8)	44.8 (26)	32.4 (11)
66+	25.0 (8)	50.0 (29)	37.8 (14)
			
Education			
<10 y	21.9 (7)	43.9 (25) **	34.4 (11)
12 yr Tafe/trade	28.1 (9)	35.1 (20)	34.5 (10)
Tertiary	50.0 (16)	21.0 (12)	28.6 (8)
			
BMI			
Healthy weight (20–24.99)	40.6 (13)	28.6 (16)	37.9 (11)*
Overweight (25–29.99)	31.3 (10)	48.2 (27)	32.4 (12)
Obese (30+)	28.1 (9)	23.2 (13)	20.7 (6)
			
Time since diagnosis			
< 4 months	35.5 (11)	33.9 (19)	26.7 (8)
5–12 months	35.5 (11)	39.3 (22)	42.4 (14)
13+ months	29.0 (9)	26.8 (15)	20.8 (5)
			
Treatment (N)			
None	0	9.4 (5)	
Surgery only	6.3 (2)	28.3 (15)	
Combination	90.6 (29)	50.9 (27)	
Other	3.1 (1)	11.3 (6)	
Missing		(6)	

* p < .05 ** p < .001; sufficient PA >150 total minutes per week

### Quality of life and depression

**Figure 1:**
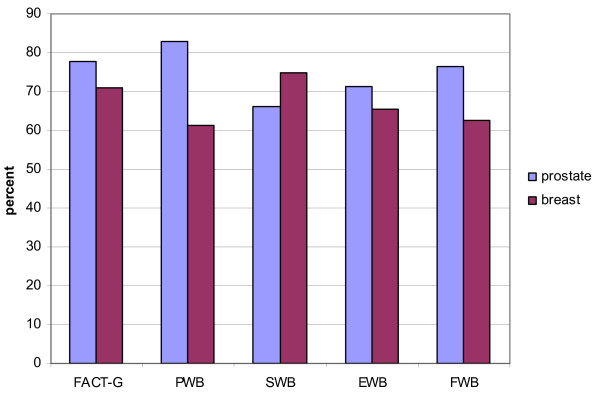
**Proportions above FACT-G cancer norms by cancer site**. PWB = physical wellbeing; EWB = emotional wellbeing; SWB = social wellbeing; FWB = functional wellbeing.

The FACT-G and CES-D data are presented in Table 2. The FACT-G total and 3 of the 4 subscale mean scores for our participants were significantly higher than the normative data for adult cancer patients reported by Brucker et al. (2005) [38]. As illustrated in Figure 1 more than 70% of our participants had FACT-G total scores and more than 60% had subscale scores that were higher than the normative means. However, in our sample 24.7% of the participants had FACT-G total scores below the normative mean; in addition, 24.7% had at a PWB score, 30.8% had a SWB or EWB score, and 28.6% had a PWB score below the relevant normative mean.

**Table 2:** Mean scores on FACT-G and CES-D

	Mean (SD)	Norms (SD)*	One-sample t-tests
FACT			
Physical wellbeing	23.4 (4.8)	21.3 (6.0)	p = .000
Emotional wellbeing	19.8 (3.4)	18.7 (4.5)	p < .01
Social wellbeing	23.2 (4.0)	22.1 (5.3)	p < .05
Functional wellbeing	21.4 (5.7)	20.9 (6.8)	ns
Breast additional	22.3 (6.8)		
Prostate additional	35.8 (5.8)		
			
FACT-G	87.8 (12.5)	80.9 (17.0)	p = .000
			
Depression	11.7 (8.4)		

* Brucker et al, 2005

Brucker et al. [38] have proposed that a FACT-G score that differs >5 points and a FACT subscale score that differs by > 2 points from the normative mean are clinically significant. In this sample 61.8% had a FACT-G score that was >5 points above the normative mean (thus clinically better QOL) while 19.1% had a FACT-G score that was >5 points below the normative mean (thus clinically worse QOL). In addition, almost one in five individuals had subscale scores that were >2 points below the mean (thus clinically worse) – FWB (28.6%), PWB (19.1%), SWB (18.7%) and EWB (17.6%).

The mean CES-D (depression) score for our participants was 11.4. Using a score of ≥16 to indicate depression, 30.8% of our sample was considered depressed with rates of 28.8% among prostate cancer participants and 34.4% among breast cancer participants.

### Change in physical activity level pre to post diagnosis

**Figure 2:**
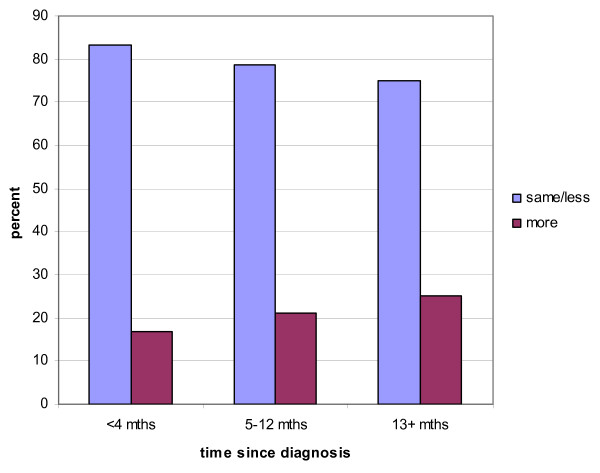
Change in physical activity by time since diagnosis.

A decline in minutes of PA per week following a cancer diagnosis occurred for total PA and for all three levels of PA intensity. Total PA declined by a mean of 72 minutes (t([1,90] = 3.71, p < .000) from pre to post diagnosis. There were also significant declines in the mean number of minutes of low PA (-53; t [1,87] = 3.01, p < .000] and moderate PA (-19; t [1,89] = 1.98, p < .05) from pre to post diagnosis. Participants reported spending little time engaging in strenuous PA in either the pre or post diagnosis periods. Change in PA level following a diagnosis was not related to time since diagnosis (Figure 2), age or education level.

**Figure 3:**
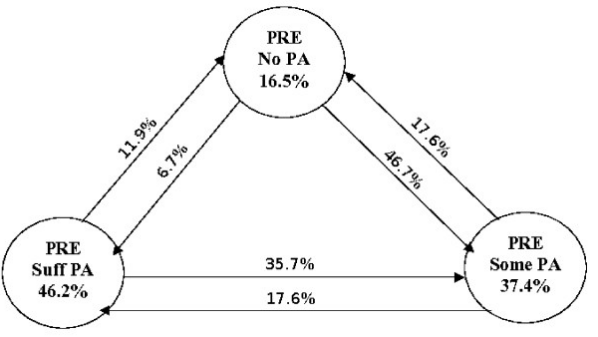
Proportion in pre diagnosis PA categories that moved to another category post diagnosis.

Chi square examination of pre and post diagnosis proportions in each PA category found significant differences (χ^2 ^= 20.8, p = .000). The proportion of participants reporting no PA increased slightly (16.5% to 19.8%) while the proportion reporting some level of PA (37.4% to 48.4%) had a relative increase of 29.4% between the pre and post time periods. The percent of participants achieving the recommended standard of >150 minutes per week of PA decreased from 46.2% to 31.9% (a relative decrease of 30.9%). Figure 3 illustrates the patterns of change for the three pre diagnosis categories of PA. Among participants who reported no PA pre diagnosis 53.4% had increased their PA levels post diagnosis with 46.7% moving into the 'some' PA category and 6.7% into the 'sufficient' PA category. On the other hand, 47.6% of participants in the 'sufficient' category at baseline decreased their PA levels post diagnosis, with 35.7% moving the 'some' category and 11.9% reporting no physical activity.

### Quality of life, depression and change in physical activity level

**Figure 4:**
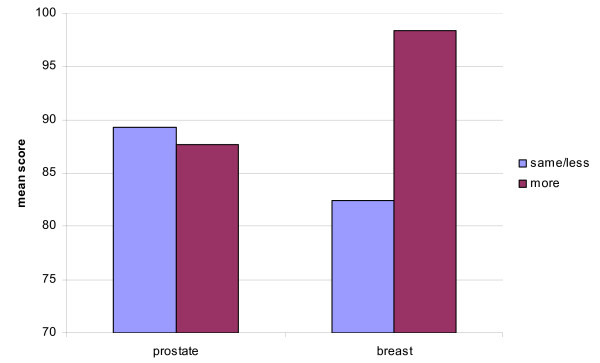
Change in physical activity by mean score on FACT-G for cancer site.

Overall 20.9% of participants reported having increased their total PA between the pre to post diagnosis periods. ANOVA was used to examine whether mean scores on FACT-G or CES-D differed between those participants who had increased PA versus those who had maintained or reduced their PA level. Breast cancer participants who had increased their PA levels reported significantly higher mean scores for the PWB subscale (26 versus 21; F[1,29] = 5.19, p < .03), the EWB subscale (22 versus 19; F[1,30] = 4.57, p < .04); the FWB subscale (26 versus 19; F[1,30] = 9.03, p < .001) and FACT-G (98 versus 82; F[1,30] = 9.13, p < .001; Figure 4). No significant differences were found for prostate cancer participants on FACT-G or subscales with change in PA. The mean CES-D scores were not significantly different between those who increased their PA levels and those whose PA levels remained the same or were reduced although a strong trend was in evidence (6 versus 14, p < .07).

### Quality of life and post diagnosis physical activity level

**Figure 5:**
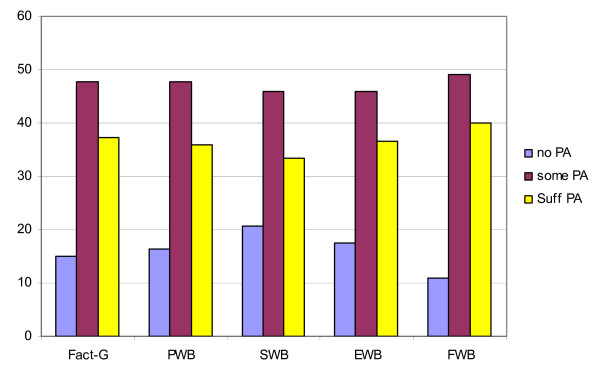
**Proportion above clinically different score on cancer norms for FACT-G (≥5) and subscales (≥2) by physical activity category**. PWB = physical wellbeing; EWB = emotional wellbeing; SWB = social wellbeing; FWB = functional wellbeing.

One way ANOVA was conducted to examine the relationship of demographic variables and PA with FACT-G and its subscales (Table 3). Pre diagnosis PA level was not found to be related to FACT-G, FACT-G subscales or CES-D scores. Significant differences in post diagnosis PA level were found for FACT-G (F[2,86] = 5.29, p < .007), Breast concerns (F[2,29] = 4.13, p < .017), PWB (F[2,87] = 3.58, p < .04) and FWB (F[2,87] = 10.01, p = .000) subscales. There were also significant differences in PWB scores by cancer site/gender (F[1,87] = 4.13, p < .04) with men reporting a significantly higher mean score (24 versus 22), and by age (F[2,87] = 3.11, p < .05) with older adults having higher PWB scores. There were no significant differences in QOL measures for time since diagnosis. As illustrated in Figure 5 participants who were in either the 'some' or 'sufficient' PA categories at post diagnosis were more likely to have FACT-G and subscale scores that differed positively from the normative means from the perspective of clinical significance. This was significant for FACT-G (χ^2 ^= 8.45, p < .02) and FWB (χ^2 ^= 16.4, p = .000) and close to significance for PWB (p < .06).

**Table 3:** Mean scores (SD) on quality of life and depression by demographics and pre and post diagnosis category of physical activity

	**FACT-G**	**Breast**	**Prostate**	**PWB**	**EWB**	**SWB**	**FWB**	**Depression**
**Prostate**	89 (12)		36 (6)	24 (4)*	20 (3)	23 (4)	22 (6)	11 (8)
**Breast**	86 (13)	22 (7)		22 (5)	19 (3)	24 (4)	20 (6)	12 (9)
								
**Age**								
<50	83 (15)	16 (7)^a^	40 (3)	21 (6)*	19 (3)	24 (5)	19 (6)	14 (10)
51–65	87 (12)	19 (5)	35 (5)	23 (5)	20 (4)	22 (4)	22 (5)	12 (8)
66+	91 (10)	21 (6)	35 (6)	25 (4)	20 (3)	24 (3)	22 (6)	10 (7)
								
**Education**								
<10 years	86 (14)	20 (10)	34 (6)^a^	23 (5)	20 (4)	22 (4)^a^	21 (6)	14 (10)^a^
12 y/TAFE/trade	89 (10)	23 (4)	37 (5)	24 (4)	20 (3)	23 (4)	21 (6)	11 (6)
Tertiary	90 (11)	23 (7)	38 (5)	24 (5)	20 (4)	25 (3)	22 (5)	9 (9)
								
**BMI**								
Healthy	91(11)*	25 (6)^a^	37 (6)^a^	24 (4)*	20 (3)	24 (4)	22 (5)	9 (7)**
O/weight	89 (13)	23 (7)	36 (6)	24 (3)	20 (3)	24 (4)	22 (6)	11 (7)
Obese	83 (14)	18 (7)	32 (5)	21 (5)	19 (4)	22 (5)	20 (6)	16 (10)
								
**PA (pre)**								
None	89 (14)	20 (6)	36 (6)	24 (5)	20 (3)	24 (3)	21 (8)	11 (9)
Some	86 (13)	23 (7)	36 (5)	23 (5)	20 (3)	23 (4)	21 (6)	12 (9)
Sufficient	89 (12)	22 (7)	35 (6)	24 (5)	20 (4)	23 (4)	22 (5)	11 (8)
								
**PA (post)**								
None	81 (13)*	18 (4)*	32 (5)^a^	21 (5)*	19 (3)	24 (3)	16 (7)**	17 (9)*
Some	88 (13)	22 (7)	37 (6)	23 (5)	20 (3)	23 (4)	22 (5)	11 (8)
Sufficient	92 (10)	29 (3)	36 (6)	25 (4)	21 (4)	23 (4)	23 (4)	9 (7)

^a ^p < .06; * p < .05; ** p < .01; higher scores on FACT scales indicate better QOL; PWB = physical wellbeing; EWB = emotional wellbeing; SWB = social wellbeing; FWB = functional wellbeing; BMI = body mass index; breast = additional items relating specifically to breast cancer; prostate = additional items relating specifically to prostate cancer; PA = physical activity

As *change *in PA demonstrated significant cancer site differences in QOL measures, we analysed post diagnosis PA results separately by cancer site. For prostate cancer participants, a significant difference in FWB scores was found (F[2,56] = 3.54, p < .04) with those who reported not taking part in any PA in the last week having significantly lower FWB scores (18.01) than those who reported having >150 minutes of PA in the last week (23.5). Among breast cancer participants, a significant difference in mean scores on FWB (F[2,29] = 5.05, p < .02), and Breast concern scores (F[2,29] = 4.68, p < .02) was demonstrated. Thus those who reported not taking part in any PA in the last week scored significantly lower on the FWB (15.3) and Breast concerns (18.1) subscales than those participants who reported having >150 minutes of PA in the last week (22.8 and 28.5 respectively).

### Depression and post diagnosis physical activity level

Depression was not found to be significantly associated with cancer site, age, education level or time since diagnosis. Depression was not related to pre diagnosis PA but was significantly related to post diagnosis PA (F[2,87] = 4.79, p < .01). Thus as the amount of PA post diagnosis increased the depression scores decreased. A significantly greater proportion of participants who reported taking part in no PA post diagnosis were identified as being depressed (55.6%) compared to participants who reported at least some level of PA in the last week (27.3%; χ^2 ^= 6.83, p < .04).

### Body mass index (BMI)

**Figure 6:**
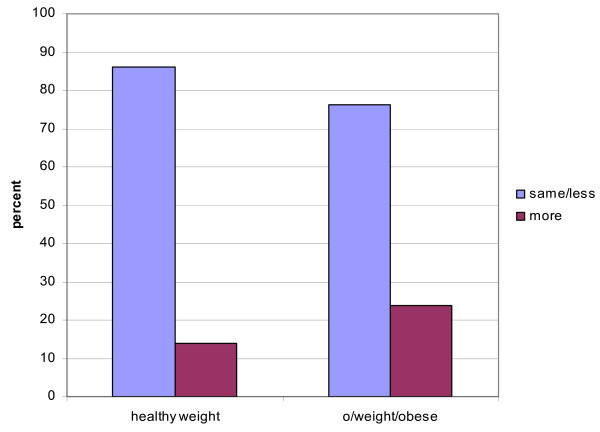
Change in physical activity by body weight category.

Chi square analysis was used to examine the relationship between BMI and current level of PA, the FACT-G QOL and CES-D depression measures. Significant differences in proportions of participants reporting no PA, some PA and >150 minutes of PA/week post diagnosis (χ^2 ^= 11.84, p = .02) were found for each BMI category, with the largest proportion of participants in the obese group being those who reported no PA (55.6%). Interestingly, there was no difference between participants with a BMI in the desired weight range versus those with a BMI reflective of being overweight or obese in terms of *changes *in PA levels from the pre to the post diagnosis period (Figure 6).

BMI was also significantly related to the FACT-G score (F[2,86] = 3.08, p < .05) and the PWB subscale score (F[2,84] = 3.45, p < .03). In general, participants scored lower on the QOL measures as BMI increased. Depression was also significantly related to BMI (F[2,85] = 4.89, p < .01) with the mean depression scores being higher in overweight and obese participants (Table 3). Participants who classified as being obese had a mean score on depression score of 17 (which is the cut-off point for depression on the CES-D scale).

### Multivariate models

Based on those relationships shown to be significant at a bivariate level, a logistic regression model was conducted to identify any predictors of change in PA. Cancer site, BMI and FACT-G were included in the model. The overall model was significant with FACT-G remaining as a significant predictor of change in PA (OR = 1.06 [1.0–1.12]). To further identify if any of the FACT-G subscales were exerting a stronger influence, a second model was run including the four subscales in addition to FACT-G. Only PWB remained significant in the model (OR = 1.44 [1.03–2.02]).

## Discussion

This study sought to extend the literature on what changes occur in physical activity (PA) levels following a cancer diagnosis and whether these changes are related to current quality of life and depressive symptoms. Overall, there was an average decline of 72 minutes of PA from pre to post diagnosis, with declines in PA occurring in all three categories of PA intensity (ie, low, moderate, strenuous). While we found an overall reduction in PA minutes, a small proportion of participants (20.9%) had increased their level of total PA between the pre and post diagnosis periods. The pattern of change differed by pre PA level. For example, among participants who were not active at pre diagnosis 53.4% reported being active in the post diagnosis period, while almost half (47.6%) of the participants who reported having a sufficient level of PA at pre diagnosis had reduced their level of PA in the post diagnosis period. Consequently they were still physically active but not active enough to yield health benefits or possibly alleviate some of the symptoms from cancer treatment. These findings are somewhat different from that of previous studies which reported reduced PA levels both during and following treatment [23-25]. These results may be attributed to the growing number of studies on exercise in the field of oncology in recent years, in particular with breast and prostate cancer patients. There could be a growing awareness among oncologists regarding physical activity and cancer treatment [39] which may be influencing oncologists to take a more favorable view of the benefits.

We found that increasing PA post diagnosis was associated with better Physical, Emotional and Functional and global QOL scores for women with breast cancer. It was expected that if a relationship was demonstrated, it would be with the Physical and Functional aspects of QOL as regular PA improves cardiovascular and muscle function. Thus the finding that increasing PA was associated with higher Emotional wellbeing scores in breast cancer participants is important. Few prior studies have used a QOL measure that included emotional wellbeing or did not report results for this component separately [40]. One study of breast cancer survivors found better EWB approached significance in women who engaged in at least one session of vigorous or moderate intensity exercise per week during treatment compared to those who did not (p < .052) [23].

This same relationship of PA and EWB was not found for prostate cancer participants. We cannot adequately explain this cancer site difference. However, questions in the FACT-G EWB subscale include coping with the illness, if they feel nervous, and if they worry about dying. It may be that men are not comfortable admitting to these types of concerns or problems. This explanation is generally consistent with the literature which reports that men score higher than women on measures of stoicism, mental and behavioral disengagement, denial and avoidance [41,42].

A second aim of this study was to explore the relationship of post diagnosis PA with QOL and depression. Post diagnosis PA was associated with better PWB, FWB and with FACT-G. Breast additional concerns were associated with current PA while Prostate additional concerns approached significance. These findings support prior studies of exercise/physical activity interventions that demonstrated improved QOL with increasing PA [43]. Almost one in three of our participants were depressed as indicated by a CES-D score of ≥16. The percent of participants who were depressed was higher among the breast cancer participants (34.4%) than the prostate cancer participants (28.8%). These results are higher than those found in studies involving ovarian cancer patients (21%) [44], and head and neck cancer patients (28%) [45], and mean scores were similar to those reported from a study involving breast cancer patients [35]. We found no difference in depression rates on time since diagnosis. This finding differs from a study involving head and neck patients in which the depression rate decreased from 28% at post diagnosis to 24% at 6 months. However, over a period of 3 years 42% of the participants were identified as being depressed suggesting that depression is a possible long term risk [45].

Participants reporting no PA had a mean depression score of 17, with all participants in this group scoring in the depressed range. This mean score is 8 points higher than the score of participants who had sufficient levels of PA at post diagnosis – a 53% relative difference. Recognition of the benefit of regular exercise/physical activity for depression is growing [46]. Intervention studies for older adults have shown greater intensity of exercise conferring a greater benefit [47]. In older adults, a 50% reduction in depression scores was achieved by 61% of those in the high intensity group as opposed to 29% in the low intensity group and 21% in the standard care group. This study supports this research by finding lower scores on depression for those participants who reported more activity.

Increased body fat has been found to be a risk factor for a range of cancers including colon, breast, endometrial and kidney [48] and weight before diagnosis has been found positively associated with breast cancer recurrence [49]. Physical activity affects body composition by promoting fat loss while preserving lean mass [50]. Obese participants in this study were the least active at pre and post diagnosis, the most depressed and reported poorer QOL. This supports prior research findings reporting a 41% decrease in sports PA amongst obese breast cancer patients compared to 24% in normal weight patients [25]. This issue warrants immediate investigation as weight gain is a problem indicated in a recent review of cancer survivors which reported 70% of breast and prostate cancer survivors being overweight or obese [51]. The nature and direction of the association of weight with PA levels is still under scrutiny. The BMI and QOL relationship needs to be interpreted with caution as these relationships also exist in the general population.

The findings from this study should be considered in light of the limitations of the study. These limitations include the retrospective measurement of pre diagnosis PA levels using a self-report measure and that we did not measure household activity. However, the instrument selected has been used in many previous studies involving cancer patients and survivors. There is always the possibility with a self-report measure that recalled information, such as PA levels, could be over or under reported. There were significant differences in age and education levels between the breast and prostate cancer survivors, with 50% of the breast cancer patients having a tertiary education. Thus applying the results of the study to wider populations needs to be done with caution. Finally, while three of the four FACT-G subscales (PWB, FWB, SWB), FACT-G and breast additional concerns had high levels of internal consistency the values for the EWB subscale and prostate additional concerns was lower than anticipated.

## Conclusion

Between one quarter and one third of the participants were identified as having emotional and/or well being problems, and more than half the participants had a PA level that was insufficient to yield expected benefits. In addition, in excess of 60% of the participants were overweight or obese. On a positive note, almost half the participants who were inactive at pre diagnosis had initiated PA by post diagnosis. What is needed from future research is a better understanding of why some participants are able to become physically active and what benefits they expect to receive from becoming active. The initial focus of investigation should involve cancer survivors who have QOL and/or depression problems. In addition, we need a better understanding of why cancer survivors decrease their levels of PA following a cancer diagnosis, and what is necessary for them to retain or increase their level of PA post diagnosis. Increasing PA to a level that is sufficient for cancer survivors to receive known benefits (eg, reduced depression, increased functional well being) should be the goal of any intervention.

## Competing interests

The author(s) declare that they have no competing interests.

## Authors' contributions

NH made significant contribution to the conception and design, acquisition of data, analysis and interpretation of data, drafting and revising of the manuscript. DCI made significant contribution to the conception, interpretation of the data, revising the manuscript critically for intellectual content.
